# Prediction of Survival and Tumor Microenvironment Infiltration Based on Pyroptosis-Related lncRNAs in Pancreatic Cancer

**DOI:** 10.1155/2022/5634887

**Published:** 2022-12-30

**Authors:** Shengbai Xue, Kangjie Shen, Kexuan Wang, Weiyu Ge, Tiebo Mao, Shumin Li, Xiaofei Zhang, Haiyan Xu, Yongchao Wang, Jiayu Yao, Ming Yue, Jingyu Ma, Yanling Wang, Daiyuan Shentu, Jiujie Cui, Liwei Wang

**Affiliations:** ^1^State Key Laboratory of Oncogenes and Related Genes, Shanghai Cancer Institute, Department of Oncology, Renji Hospital, Shanghai Jiaotong University School of Medicine, Shanghai, China; ^2^Department of Plastic Surgery, Zhongshan Hospital, Fudan University, Shanghai, China; ^3^Department of Nursing, School of Nursing, Xuzhou Medical University, Xuzhou, China

## Abstract

Pancreatic cancer (PC) is a fatal tumor with high mortality. Pyroptosis plays a tumor suppressor role as a novel cell death. However, the influences of the pyroptosis-related lncRNAs (PRlncRNAs) on the prognosis and tumor microenvironment (TME) infiltration have not been fully studied in PC. Using coexpression analysis and univariate Cox regression analysis, we identified seventeen prognostic PRlncRNAs from The Cancer Genome Atlas (TCGA) dataset, which were all expressed differently in normal and tumor samples. A seven-PRlncRNA risk signature was constructed and validated using the least absolute shrinkage and selection operator (LASSO) regression. Furthermore, we verified its independence and created a nomogram to validate the clinical viability of the risk signature. We then identified its relationship with clinical factors and evaluated its values in TME infiltration, functional enrichment, tumor mutation, and therapeutic responses in PC. Lower ImmuneScore, ESTIMATEScore, and advanced tumor stage were connected with high-risk score. The low-risk group was characterized by better OS, elevated immune activation, and higher susceptibility of pazopanib and sunitinib. The high-risk group possessed a worse immune infiltration and poor survival, with higher tumor mutations and lapatinib and paclitaxel that may be better choices in this group. In conclusion, we developed an original seven-PRlncRNA risk signature to predict prognosis, TME infiltration, tumor mutation, and therapeutic options for PC patients.

## 1. Introduction

Pancreatic cancer (PC), characterized by late diagnose, early metastasis, and poor prognosis, has become the fourth-leading cause of cancer-related death globally, projected to be the second most common cause by 2030 in the US [1]. Despite huge advances in treatment, its five-year overall survival (OS) is still under 10% [2]. Late diagnose leads to most patients losing the chance of surgery, which seemed as the only potential way of cure [3]. Recently, treatment of PC has entered the era of precision, such as targeted therapy and immunotherapy. Nevertheless, the therapeutic effects remain poor due to the lack of targets and drug resistances. Consequently, identifying novel biomarkers is urgently needed to predict survival outcome and make therapeutic decisions of PC.

Pyroptosis is a newly discovered category of programmed cell death. Under a microscope, it was described as cell swelling and bubble formation [4]. Activated by inflammasomes, caspase-1/4/5/11 triggers pyroptosis via cleaving gasdermin D, resulting in a pore which destroys the osmotic integrity of plasma membrane [5]. Unlike apoptosis, pyroptosis is lytic, with nucleus integrated and without PARP cleaving [6]. It is an important proinflammatory cell death mode involved in bacterial infection [7]. Pyroptosis is also observed in cancers. MST1 represses the progression of PC via pyroptosis induced by reactive oxygen species, independent from Hippo pathway [8]. Besides, immune checkpoint inhibitors (ICIs) have also been proved to be efficient with the concomitant induction of pyroptosis in “cold” tumor [9]. Due to these, an increasing number of prognosis signatures with pyroptosis-related genes (PRGs) have been validated to forecast long-term outcomes of PC patients.

Long noncoding RNAs (lncRNAs) emerge as significant players in regulations of transcription and translation of mRNA [10]. Longer than two hundred bases, although lncRNAs do not participate in encoding proteins, they play a vital role in PC patients. Zhou et al. have proved that lncRNA PVT1 leads to higher gemcitabine resistance in PC by activation of autophagy and Wnt/*β*-catenin axis [11]. PC progression can also be promoted by lncRNA PMSB8-AS1 via upregulating STAT1 [12]. In previous studies, m6a-related [13] and ferroptosis-related [14] lncRNAs have shown the unique ability of predicting prognosis of PC. Nevertheless, the association of pyroptosis-related lncRNAs (PRlncRNAs) with prognosis is still unclear in PC. Therefore, we aimed to explore potential pyroptosis-related lncRNAs with prognostic abilities and establish a novel signature to predict the prognoses and TME conditions of PC patients in this study.

## 2. Materials and Methods

### 2.1. Public Datasets

Transcriptome expression lists as well as clinicopathological factors of patients (*n* = 177) with PC were acquired from The Cancer Genome Atlas (TCGA) dataset. Gene IDs were transferred to gene symbols and merged with clinical features by Perl software. The excluded criteria were patients with absent information or lacking expression lists. To extract pyroptosis-related gene, we used keyword “Pyroptosis” to achieve gene list from GeneCards. Combined with the previous research [15], 121 PRGs were finally included in our study (Table S1).

### 2.2. Identification of Pytoptosis-Related lncRNAs with Predictive Abilities

For identification of PRlncRNAs, we used Pearson correlation analysis with the standards of *p* < 0.001 as well as |*R*| > 0.4. lncRNA annotation files were achieved from the human reference genome website. Moreover, we implemented univariate Cox regression analysis to choose prognostic PRlncRNAs from TCGA database with the significant level of *p* < 0.001. Differential analysis was implemented to investigate whether these PRlncRNAs expressed differently between tumor samples and nontumor samples in TCGA by R package “limma”.

### 2.3. Construction and Verification of PRlncRNA Prognostic Risk Signature

We firstly separated PC patients from TCGA database following a one-to-one ratio randomly into training set and test set. To establish and verify a PRlncRNA prognostic signature, least absolute shrinkage and selection operator (LASSO) regression analysis was taken to identify the most prognostic PRlncRNAs. We use the following Equation (1) to calculat each patient's risk score:

(1) $$\begin{array}{c} \text{risk score}=e^{\sum \text{c}\text{oefficient}\times \text{PRlncRNA expression}}. \end{array}$$

Based on the median score in training set, PC patients were separated into low-risk or high-risk subgroup. Survival analysis was then adopted with the help of R package “survival” to explore whether patients from different groups possessed distinguishing survival outcomes. Receiver operating characteristic (ROC) curves were performed by the R package “survivalROC” to verify the precision of our signature at 1, 2, and 3 years. Afterwards, we analyzed in test and entire sets to verify these results.

### 2.4. Relationship between Risk Score and Clinicopathological Features

Univariate and multivariate Cox regression analyses were implemented to certify the independent predictive ability of our PRlncRNA signature based on the entire TCGA cohort. The standard of independence was *p* < 0.05 in both analyses. We then established a nomogram combining risk score with clinical factors for survival prediction. Calibration curves of 1, 2, and 3 years were used to verify its predictive value. Besides, we investigated the association of risk score with clinical features based on TCGA cohort. Comparison of OS determined by risk score was then performed in each clinical subgroup (age, gender, grade, stage, tumor stage, and node stage) to validate the predictive value of our signature.

### 2.5. Analysis of Immune Infiltrations in Different Groups

To further verify PRlncRNAs' influence, we analyzed immune infiltrations of patients based on the entire TCGA cohort. Main types of immune cell were achieved using “CIBERSORT R script v1.03”. We then assessed the immune infiltrations in different risk groups. Besides, we used R package “limma” to investigate the association of immune checkpoint genes with PRlncRNAs and risk score.

### 2.6. Functional and Signal Pathways Affected by PRlncRNAs

To identify the potential functions among low-risk group and high-risk group, differentially expressed genes (DEGs) between subgroups were generated based on the entire TCGA cohort (|log2FC| ≥ 1 and FDR < 0.05). To investigate the potential pathways these DEGs involved in, we implemented Gene Ontology (GO) and Kyoto Encyclopedia of Genes and Genomes (KEGG) enrichment analyses with the criteria of *p* < 0.05, using the R package “clusterProfiler”. To further detect functional pathways related to two subgroups, Gene set enrichment analysis (GSEA) was employed. Significant criteria of GSEA were defined as *p* < 0.05 as well as |NES| > 1. Finally, we took Gene Set Variation Analysis (GSVA) to explore the association of risk score with functional pathways, and the result was visualized by R packages “reshape2” and “ggplot”.

### 2.7. Association of Risk Score with Tumor Mutation and Therapeutic Sensitivities

PC tumor mutation data was also acquired from TCGA dataset. Using the R package “maftools”, top 20 mutated genes in two subgroups were compared. To evaluate the risk signature's predictive value for clinical treatment of PC, with the help of R package “pRRophetic”, we explored several therapeutic half inhibitory concentrations (IC50) in two subgroups.

### 2.8. Statistical Analysis

Perl and R software were used to process and analyze data, respectively. *p* < 0.05 was seemed as significant. Pearson correlation analysis was adopted for coexpression analysis. The accuracy of our signature was tested by ROC curves. Cox regression analyses were used to identify independently prognostic factors. Different survival outcomes were investigated by log-rank test and displayed by Kaplan-Meier curve.

## 3. Result

### 3.1. Exploration of Prognostic PRlncRNAs

**Figure 1:**
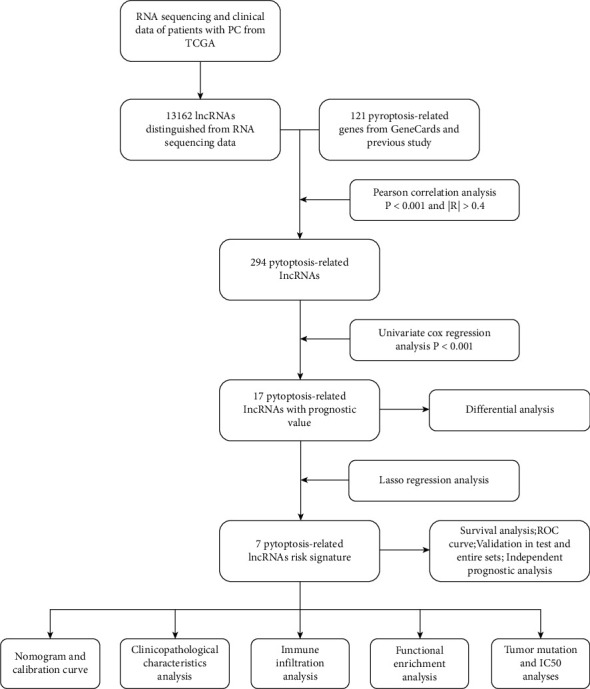
Study route.

**Figure 2:**
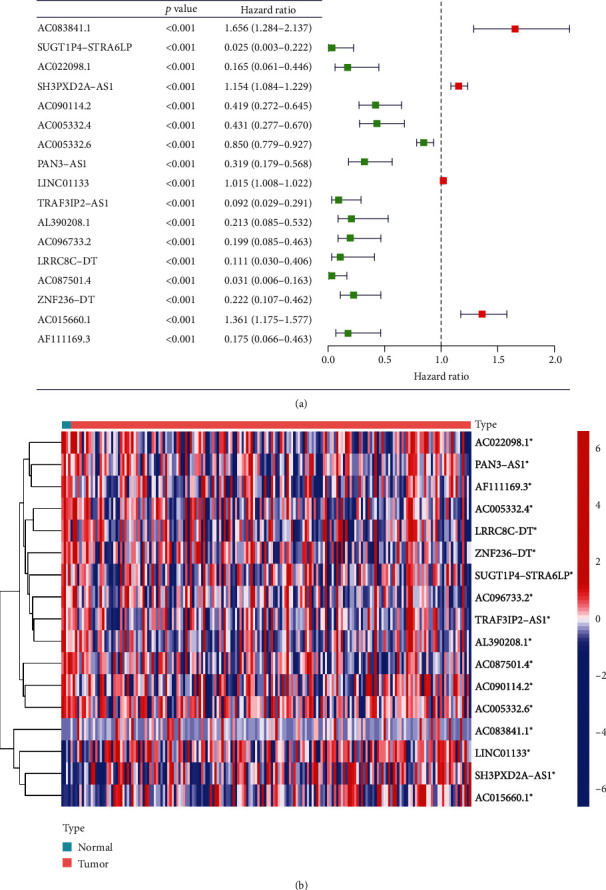
Exploration of prognostic PRlncRNAs. (a) PRlncRNAs with different hazard ratios. (b) Differential expression of prognostic PRlncRNAs among normal samples and tumor samples. PRlncRNAs: pyroptosis-related lncRNAs. ^∗^*p* < 0.05, ^∗∗^*p* < 0.01, and ^∗∗∗^*p* < 0.001.

The route of our study was concluded in Figure 1. In total, 121 PRGs and 13162 lncRNA expression profiles of PC patients were acquired from TCGA dataset as well as relevant clinical signatures (Table 1, *n* = 177). We implemented Pearson correlation analysis, and 294 PRlncRNAs were obtained for univariate Cox regression analysis (Table S2). Finally, we identified 17 PRlncRNAs with prognostic value (Figure 2(a)). AC083841.1, LINC01133, SH3PXD2A-AS1, and AC015660.1 were highly expressed in tumor samples, while AC022098.1, PAN3-AS1, AF111169.3, AC005332.4, LRRC8C-DT, ZNF236-DT, SUGT1P4-STRA6LP, AC096733.2, TRAF3IP2-AS1, AL390208.1, AC087501.4, AC090114.2, and AC005332.6 were mainly expressed in normal samples (Figure 2(b)).

### 3.2. Construction and Validation of a PRlncRNA Risk Signature

**Figure 3:**
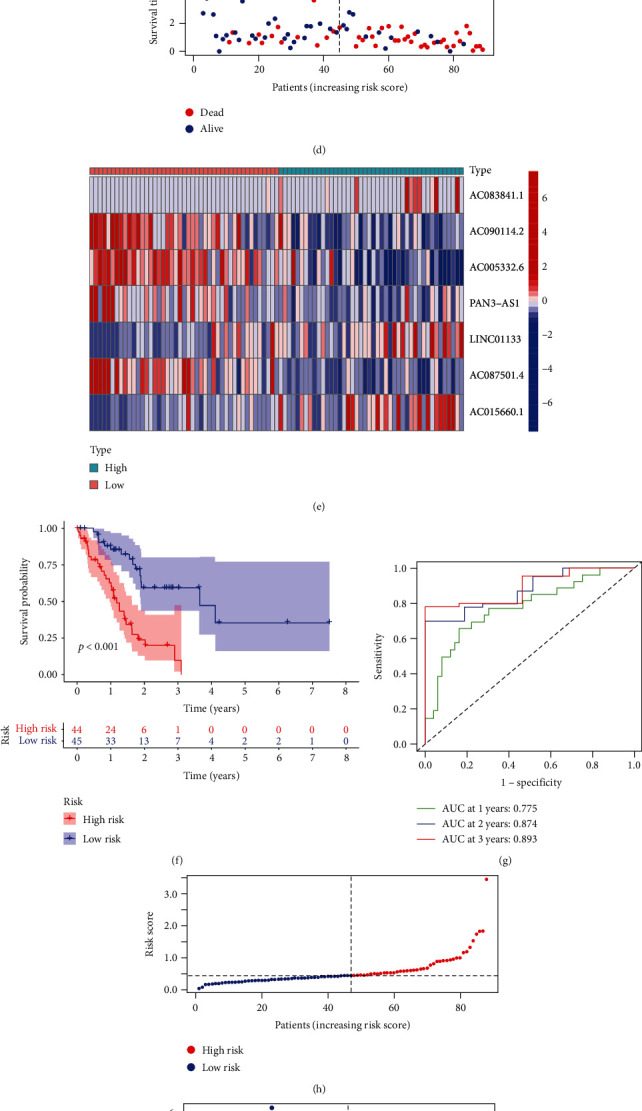
Generation and verification of the PRlncRNA signature. (a, b) Selection of seven most prognostic PRlncRNAs. (c) Grouping of PC patients in training set. (d) Survival states of PC patients from training set. (e) Visualization of 3 dangerous and 4 protective PRlncRNAs. (f) Low-risk patients possess better OS in training set. (g) Prognostic accuracy of the risk signature at first three years. (h–l) Validation by repeating analyses in test set. PRlncRNA: pyroptosis-related lncRNA; PC: pancreatic cancer; OS: overall survival.

Based on TCGA cohort, patients with PC were classified into training (*n* = 89) and test (*n* = 88) sets randomly. Seventeen prognostic PRlncRNAs were then incorporated into LASSO regression analysis in training set (Figures 3(a)–3(b)). Finally, 7 PRlncRNAs involved in risk signature were generated (Table S3). Risk score was computed using the mentioned method, and patients from training set were separated into low-risk group and high-risk group by the median of their risk scores (Figure 3(c)). Different survival states of PC patients with different risk scores were shown in Figure 3(d), while the heat map further displayed the different expression of 7 PRlncRNAs in 2 subgroups (Figure 3(e)). High-risk group was significantly related to a worse survival outcome (Figures 3(f), *p* < 0.001). The predictive veracity of our signature at 1, 2, and 3 years was validated by ROC curves (Figure 3(g)). The results above were also verified in the test set (Figures 3(h)–3(l)) and entire set (Figure S1).

### 3.3. Independence Analysis and Construction of a Nomogram

**Figure 4:**
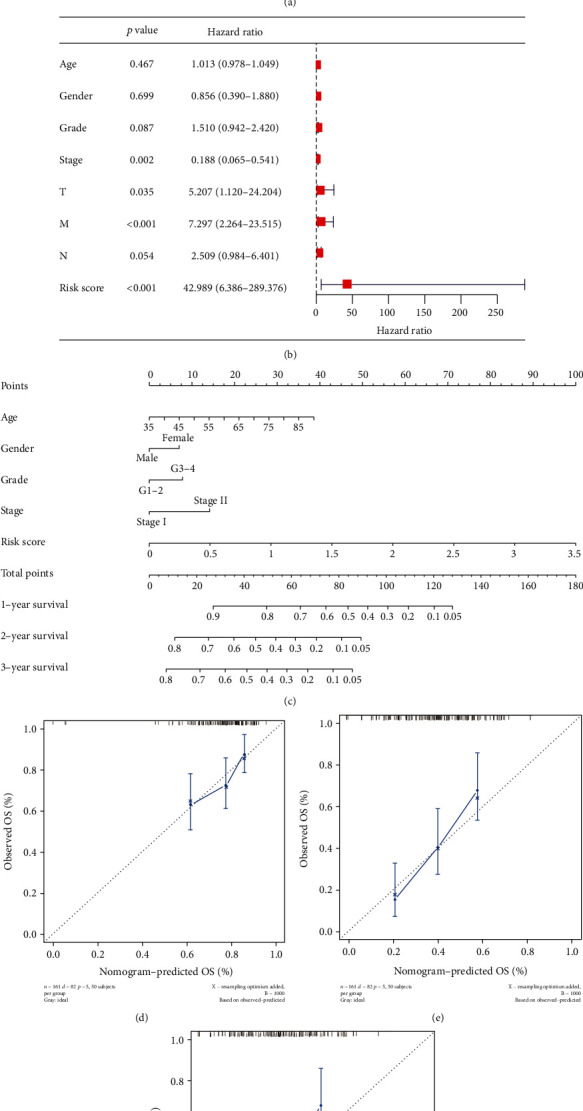
Independent analyses and establishment of a nomogram. (a, b) Univariate and multivariate Cox regression analyses. (c) Nomogram. (d–f) Calibration curves at first three years.

We evaluated whether our risk score possessed prognostic independence based on the entire TCGA cohort. Its prognostic independence was verified by univariate (Figure 4(a), HR: 8.177, 95% CI: 2.433–27.480, *p* < 0.001) and multivariate Cox regression analyses (Figure 4(b), HR: 42.989, 95% CI: 6.386–289.376, *P* < 0.001). Several clinical factors along with the risk score were brought into a nomogram to predict survival outcomes of PC patients (Figure 4(c)). We excluded patients in stage III-IV which lead to an inaccurate result due to their few numbers. Furthermore, calibration curves were plotted for validating its accuracy (Figures 4(d)–4(f)). Overall, the prognostic value of our nomogram is stable.

### 3.4. Relationships between Risk Score and Clinicopathological Features

**Figure 5:**
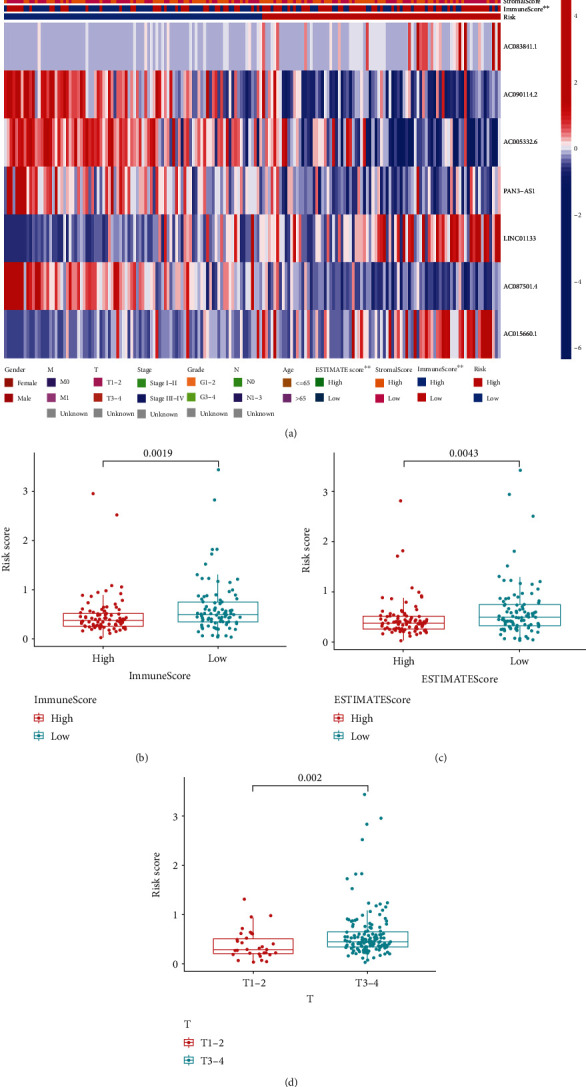
Connection between risk score and clinical characteristics. (a) Heat map visualizes the correlation of risk score with clinical factors. (b–d) Risk score in different groups of ImmuneScore, ESTIMATEScore, and T stage. ^∗^*p* < 0.05, ^∗∗^*p* < 0.01, and ^∗∗∗^*p* < 0.001.

Based on the entire TCGA database, a heat map illustrated associations of PRlncRNAs expression, risk score with clinicopathological factors (Figure 5(a)). As shown, ImmuneScore and ESTIMATEScore displayed significant differences between two risk groups. For further verification, we evaluated difference of risk score among different clinical characteristics in detail. As we predicted, higher risk scores were retained in low immune score subgroup and low estimate score subgroup (Figures 5(b)–5(c), *p* < 0.01). Interestingly, patients with T1-2 had lower risk scores (Figure 5(d), *p* < 0.01). We further performed survival analyses between clinical subgroups. The subgroup survival analyses validated the value of the risk signature for different clinical characteristics (Figure S2).

### 3.5. Immune Infiltration Analyses

**Figure 6:**
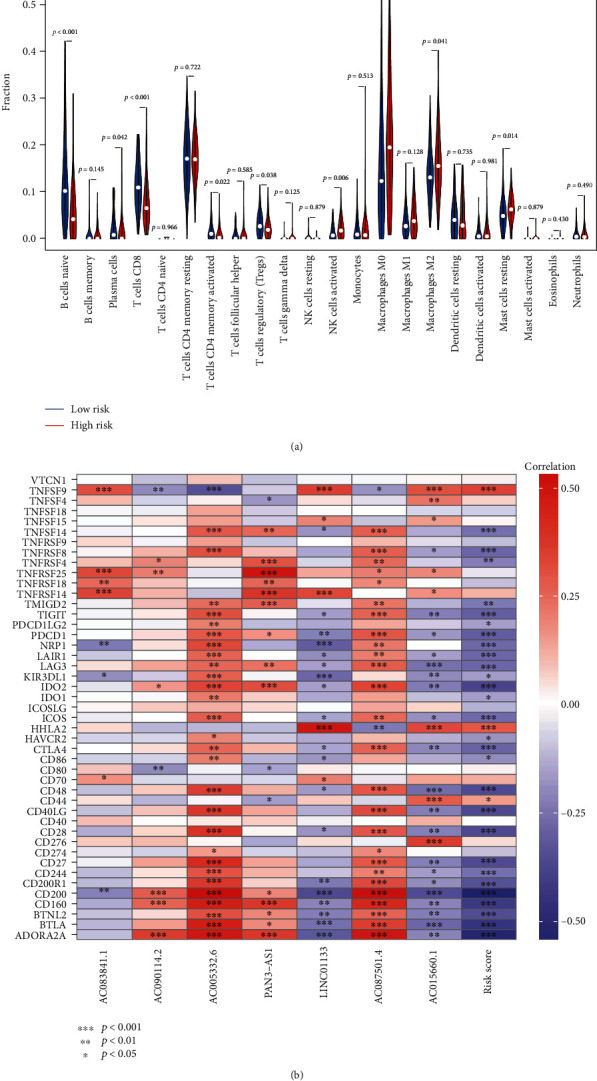
Immune infiltrations in two risk groups. (a) Immune landscapes of two risk groups. (b) Association of immune check point genes with risk score and PRlncRNAs. PRlncRNAs: pyroptosis-related lncRNAs; ^∗^*p* < 0.05, ^∗∗^*p* < 0.01, and ^∗∗∗^*p* < 0.001.

We explored the immune infiltration conditions among two risk groups due to their significantly different ImmuneScore. As results, the infiltration level of naive B cell, plasma cell, CD8 T cell, activated memory CD4 T cell, and regulatory T cell increased in low-risk group, while activated NK cell, M0 macrophage, M2 macrophage, and resting mast cell were raised in high-risk group (Figure 6(a)). For details, association of risk score with immune infiltration was explored. Activated NK cell (Figure S3a, *p* < 0.001), M0 macrophage (Figure S3b, *p* < 0.01), M1 macrophage (Figure S3v, *p* < 0.01), M2 macrophage (Figure S3d, *p* < 0.01), and resting mast cell (Figure S3e, *p* < 0.01) were positive correlated with risk score, while naive B cell (Figure S3f, *p* < 0.001), plasma cell (Figure S3g, *p* < 0.01), CD8 T cell (Figure S3h, *p* < 0.001), activated memory CD4 T cell (Figure S3i, *p* < 0.01), regulatory T cell (Figure S3j, *p* < 0.05), and gamma delta T cell (Figure S3k, *p* < 0.05) remained negative correlation. Taken together, PC patients with low-risk scores possessed better immune infiltrations. Besides, the association of immune checkpoint gene expressions with risk score and PRlncRNAs was further investigated. Most of genes were expressed negatively with risk score (Figure 6(b)).

### 3.6. Correlation of Signal and Functional Pathways with PRlncRNAs

**Figure 7:**
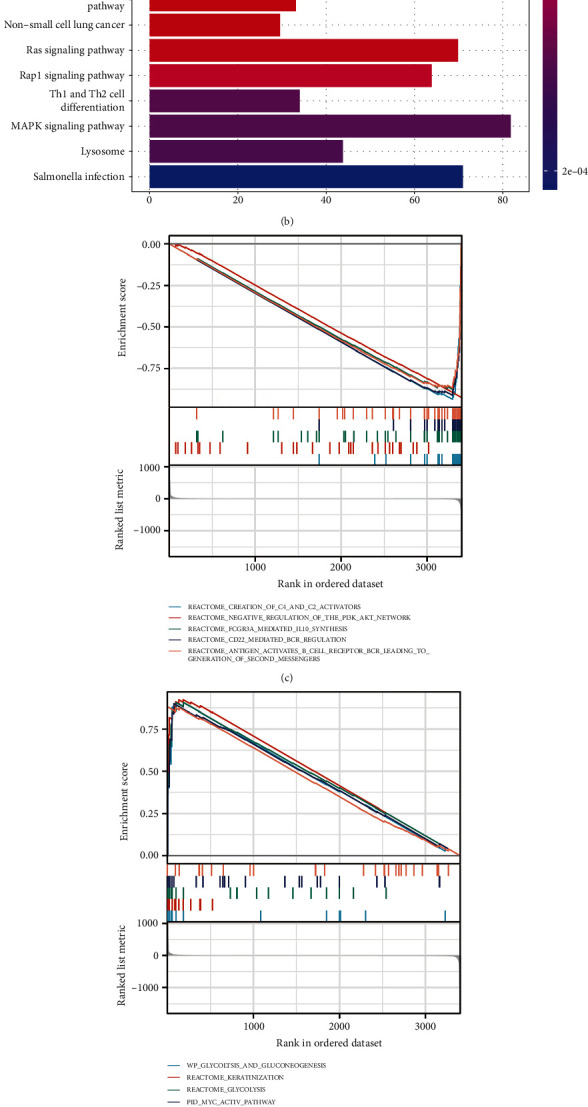
Functional enrichment affected by PRlncRNAs. (a) GO function enrichment in risk signature. (b) KEGG pathway enrichment in risk signature. (c) GSEA performed in low-risk group. (d) GSEA performed in high-risk group. (e) GSVA performed in the risk signature. PRlncRNAs: pyroptosis-related lncRNAs; GO: Gene Ontology; KEGG: Kyoto Encyclopedia of Genes and Genomes; GSEA: gene set enrichment analysis; GSVA: gene set variation analysis; ^∗^*p* < 0.05, ^∗∗^*p* < 0.01, and ^∗∗∗^*p* < 0.001.

Moreover, we explored DEGs among two risk groups and performed enrichment analysis to explore potential signal pathways which these DEGs involved in. GO analysis illustrated that DEGs primarily took part in immune responses like T cell activation, leukocyte proliferation, and immune response-activating signaling pathways (Figure 7(a)). KEGG analysis also indicated that these genes were involved in B cell receptor signaling pathway. Besides, they were also enriched in MAPK and RAS signaling pathway, which played crucial roles in tumorigenesis of PC (Figure 7(b)). GSEA showed several pathways of immune and tumor suppression that were correlated with low-risk group (Figure 7(c)), while “Glycolysis” was found to notably activate in high-risk group (Figure 7(d)). Several results have also been verified in GSVA, such as “Hypoxia” and “Glycolysis,” which have been verified correlated with tumor progression, and have positive associations with risk score (Figure 7(e)).

### 3.7. Analyses of Tumor Mutation and IC50 between Risk Subgroups

**Figure 8:**
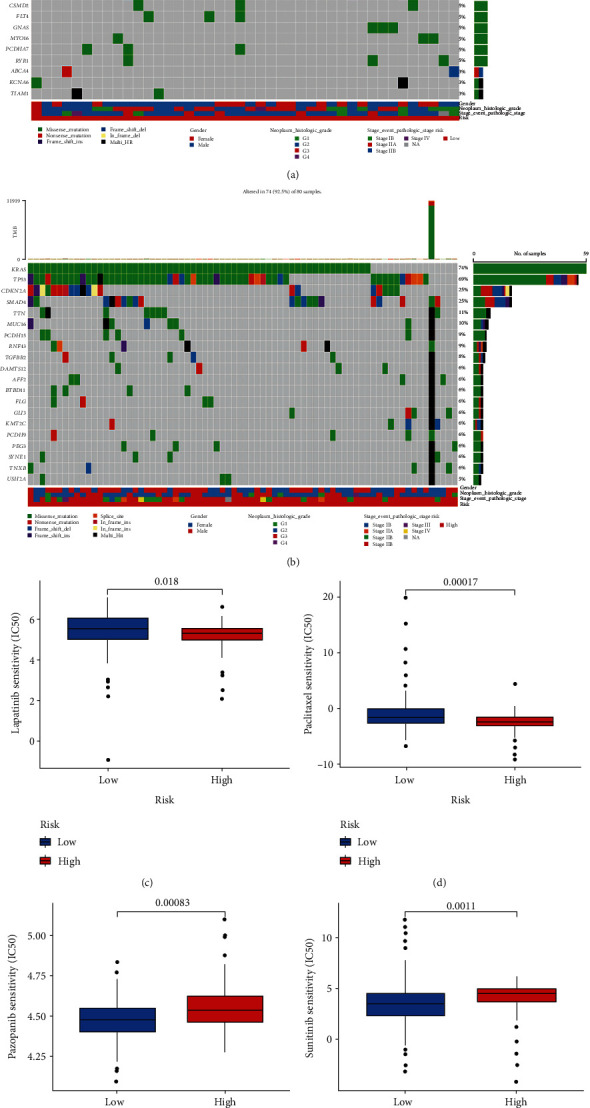
Analyses of tumor mutation and IC50 between two risk groups. (a, b) Oncoplots displayed top 20 mutated genes in two subgroups. Chemosensitivities of (c) lapatinib, (d) paclitaxel, (e) pazopanib, and (f) sunitinib in two risk subgroups. IC50: half inhibitory concentration.

Oncoplots showed that high-risk subgroup possessed increased genetic mutations (Figures 8(a)–8(b)). IC50 analyses demonstrated the prediction of several drugs with different effects in two groups. Higher sensitivities to lapatinib and paclitaxel (Figures 8(c)–8(d), *p* < 0.05) were demonstrated in high-risk group, whereas pazopanib and sunitinib (Figures 8(e)–8(f), *p* < 0.01) were possibly better choices for low-risk patients.

## 4. Discussion

PC remains a deadliest type of malignancies, considered as the king of cancer with 5-year OS less than 10% [16]. A variety of treatments has been attempted, but the prognosis of patient remains worse. The development of RNA-targeted therapies establishes a platform for drug discovery [17]. Thus, finding small molecules that can be targeted is crucial. Pyroptosis, as a new death category in cells, has shown its potentiality of being a therapeutic target in cancer [18]. Cannabidiol can suppress hepatocellular carcinoma via GSDME dependent pyroptosis [19]. Moreover, damage-associated molecular patterns (DAMPs) releasing induced via pyroptotic cell death enhance the efficiency of immunotherapy such as ICIs [20]. lncRNAs participate in various cell processes in tumor, including pyroptosis. In gastric cancer, lncRNA ADAMTS9-AS2 not only suppressed tumor cell but also increased chemosensitivity of cisplatin through pyroptosis mediated by NLRP3 [21]. Contrarily, ovarian cancer has progressed due to inhibition of pyroptosis induced by lncRNA HOTTIP [22]. However, how PRlncRNAs influence PC is still uncertain. In this study, we explored prognostic PRlncRNAs and constructed a risk signature based on TCGA dataset. We also investigated their effect on TME infiltration.

A total of 294 PRlncRNAs were obtained by Pearson correlation analysis. We then performed univariate Cox regression analysis and ended up with 17 prognostic PRlncRNAs. All of them were expressed differently between nontumor and tumor samples. Through LASSO regression, seven-PRlncRNA (AC083841.1, AC090114.2, AC005332.6, PAN3-AS1, LINC01133, AC087501.4, and AC015660.1) risk signature was established in training set. AC083841.1, LINC01133, and AC015660.1 were considered as dangerous lncRNAs, while others were protective. Patients were divided by median of risk scores (computed by e ^ (0.324∗AC083841.1 − 0.514∗AC090114.2 − 0.005∗AC005332.6 − 0.076∗PAN3 − AS1 + 0.010∗LINC01133 − 1.404∗AC087501.4 + 0.202∗AC015660.1)). Patients with high-risk scores had terrible outcomes. In terms of ROC curves, our risk signature was accurate at 1, 2, and 3 year based on AUCs of 0.775, 0.874, and 0.893, respectively. To test its validation furtherly, we repeated all analyses in test set and entire set. Overall, our risk signature had a stable prognostic ability.

Among these PRlncRNAs, lncRNA LINC01133 has been mentioned several times in pancreatic cancer studies. LINC01133 was upregulated in PC, accelerating the progression of tumor cells [23]. Liu et al. proved that LINC01133 promoted precancerous lesions in PC through Wnt/*β*-catenin axis by silencing AXIN2 [24]. As discovered by Huang et al., C/EBP*β*-LINC01133 played a crucial role in enhancing proliferation of PC through upregulation of CCNG1 [25]. Zhang et al. also suggested that LINC01133 sponged miR-216a-5p to trigger tumorigenesis in PC [26]. Taken together, these previous studies were consistent with our result that LINC01133 was a high-risk factor in PC.

Furthermore, we adopted Cox regression analyses to confirm the independence of PRlncRNA risk signature. As a result, it was verified as an independent prognostic factor. A nomogram used in clinic incorporating risk score with other clinical factors was then established. Stage III-IV patients were too few to participate in analysis, thus deleted (*n* = 7 of 177). Through the verification of calibration curves, our nomogram had a great prognostic ability for PC patients at 1, 2, and 3 years. Next, we investigated the connection between risk score and clinical characteristics. Lower ImmuneScore and lower ESTIMATEScore were relevant to higher risk scores. Besides, patients at terminal tumor stage also possessed higher risk scores. Survival analyses proved the risk score could predict patients' survival status in different clinical subgroups, which low-risk patients' outcomes were better.

Different cells were infiltrated in TME, which were relevance to prognosis and effect of immunotherapy. Thus, we analyzed the immune infiltration among different groups. Low-risk group was mainly filled with immune attack cells, such as CD8 T cell, which may explain its better OS. M0, M2 macrophages, and resting mast cell were highly infiltrated in high-risk group, contributing to an immunosuppressive microenvironment. Exosomes derived from M2 macrophages have been proved to accelerate angiogenesis and development of PC via targeting E2F2 [27]. In preclinical PC model, mast cells released TGF-*β*, thus activating SMAD4 signal transduction and developing resistance to first-line chemotherapeutics [28]. There were several immune check point gene expressions related to risk score and PRlncRNAs, such as TNFSF9, CTLA4, and CD44. CTLA4 was proved to be a potential therapeutic target in PC [29]. CD44 was expressed higher in several tumors including PC, also described as an important target [30]. Their correlations with PRlncRNA and risk score may offer potential suggestions to clinical treatment for PC patients based on their risk scores.

To explore potentially functional enrichment among two groups, we implemented GO and KEGG analyses. DEGs among two risk groups were primarily enriched in immune processes, indicating that pyroptosis had influences on the tumor immune microenvironment. In addition, they were related to MAPK, RAS, and Rap1 signaling pathways. RAS was an oncogene promoting tumorigenesis, and KRAS mutation was found in 90% of patients with PC [31]. Invasion and metastasis of PC were facilitated through EGFR signal pathway activated by Rap1 [32]. GSEA depicted that low-risk group was mainly relevant to immune-related pathways. Synthesis of IL10 simultaneously enhanced antitumor immunity and inhibited tumor-associated inflammatory [33]. Besides, negative regulation of PI3K/AKT pathway [34] and creation of C2 and C4 activators [35, 36] had also been suggested to play anti-PC roles. Association of “Glycolysis” with high-risk group was significant. As part of the metabolic reprogramming, glycolysis played a crucial role in regulating metastasis of PC [37]. This association was also found in GSVA. Besides, “Hypoxia” was also related to the risk score positively, which served as a promotion of PC progression [38]. These relationships between functional pathways and risk score may help us better understand the biological processes in PC affected by PRlncRNAs.

Accumulation of tumor mutations triggered the malignant transformation in PC [39]. Therefore, analysis of tumor mutation was performed among two subgroups. Patients in high-risk group possessed higher genetic mutations, especially KRAS, TP53, CDKN2A, and SMAD4, which related to the tumorigenesis of PC [40]. Moreover, we investigated the predictive value of the PRlncRNA signature for sensitivities of medicines. Patients in different groups were predicted to show sensitivities to different therapeutics, illustrating the risk signature could provide treatment options for PC patients. Since the majority of these drugs were targeted, the clinical use should also follow the results of gene sequencing.

Our study still existed on several limitations. First of all, there was no external validation of our results in other datasets due to the lack of lncRNA expression profiles. Secondly, our results of analyses have not been verified by experiments. We hope to address these two issues to improve our PRlncRNA signature in the future.

## 5. Conclusions

We identified a novel seven-PRlncRNA risk signature for pancreatic cancer. Risk score generated from the signature acted as an independent prognostic factor to predict long-term outcomes, TME infiltration, functional pathways, tumor mutation, and medical sensitivities of PC patients from TCGA cohort. This research may provide a new view for the clinical treatment of PC.

## Figures and Tables

**Table 1:** Clinical characteristics of pancreatic cancer patients from TCGA.

Clinical variables	Number	%
Total	177	100
Age		
≤ 65y	93	52.54
> 65y	84	47.46
Gender		
Male	97	54.80
Female	80	45.20
Grade		
1-2	125	70.62
3-4	50	28.25
X	2	1.13
Stage		
I-II	167	94.35
III-IV	7	3.96
Unknown	3	1.69
Tumor classification		
T1-2	31	17.51
T3-4	144	81.36
Tx	2	1.13
Node classification		
N0	49	27.68
N1-3	123	69.49
Nx	5	2.83
Metastasis classification		
M0	79	44.63
M1	4	2.26
Mx	94	53.11
Survival time		
≤ 3y	161	90.96
> 3y	16	9.04
Survival status		
Survival	89	50.28
Death	88	49.72

## Data Availability

The datasets (TCGA) for this study can be found in https://portal.gdc.cancer.gov. The datasets (GeneCards) for this study can be found in https://www.genecards.org.

## Supplementary Materials

Supplementary Materials Figure S1: verification of the risk signature in entire set. (a) Grouping. (b) Scatter plot. (c) Heat map. (d) Survival analysis. (e) ROC curves. ROC: receiver operating characteristic. Figure S2: survival analyses of clinical subgroups. (a) Age ≤ 65. (b) Age > 65. (c) Female. (d) Male. (e) Grade 1-2. (f) Grade 3-4. (g) Stage I-II. (h) Stage III-IV. (i) T1-2. (j) T3-4. (k) N0. (l) N1-3. Figure S3: correlation of risk score with immune cells. (a) Activated NK cell. (b) M0 macrophage. (c) M1 macrophage. (d) M2 macrophage. (e) Resting mast cell. (f) Naive B cell. (g) Plasma cell. (h) CD8 T cell. (i) Activated memory CD4 T cell. (j) Regulatory T cell. (k) Gamma delta T cell. Table S1: gene list of 121 pyroptosis-related genes. Table S2: a total of 294 pyroptosis-related lncRNAs in TCGA. Table S3: seven pyroptosis-related lncRNAs involved in the risk signature.
