# Supplementary material for: Prediction of Survival and Tumor Microenvironment Infiltration Based on Pyroptosis-Related lncRNAs in Pancreatic Cancer
Source: Dis Markers. 2022 Dec 30;2022:5634887. doi: 10.1155/2022/5634887 (PMC9822759; doi:10.1155/2022/5634887)
Supplement: Supplementary Materials — Figure S1: verification of the risk signature in entire set. (a) Grouping. (b) Scatter plot. (c) Heat map. (d) Survival analysis. (e) ROC curves. ROC: receiver operating characteristic. Figure S2: survival analyses of clinical subgroups. (a) Age ≤ 65. (b) Age > 65. (c) Female. (d) Male. (e) Grade 1-2. (f) Grade 3-4. (g) Stage I-II. (h) Stage III-IV. (i) T1-2. (j) T3-4. (k) N0. (l) N1-3. Figure S3: correlation of risk score with immune cells. (a) Activated NK cell. (b) M0 macrophage. (c) M1 macrophage. (d) M2 macrophage. (e) Resting mast cell. (f) Naive B cell. (g) Plasma cell. (h) CD8 T cell. (i) Activated memory CD4 T cell. (j) Regulatory T cell. (k) Gamma delta T cell. Table S1: gene list of 121 pyroptosis-related genes. Table S2: a total of 294 pyroptosis-related lncRNAs in TCGA. Table S3: seven pyroptosis-related lncRNAs involved in the risk signature. [file 5634887.f1.zip › Table S1.docx]

Table S1： Gene list of 121 pyroptosis-related genes.

| Gene | Category | GIFtS | GC id | Score |
| --- | --- | --- | --- | --- |
| HNP1 | Genetic Locus | 1 | GC09U900671 | 2.36 |
| GSDMD | Protein Coding | 41 | GC08P143553 | 21.76 |
| GSDME | Protein Coding | 32 | GC07M024699 | 15.85 |
| NLRP3 | Protein Coding | 48 | GC01P247415 | 15.22 |
| CASP1 | Protein Coding | 51 | GC11M105025 | 13.17 |
| CASP4 | Protein Coding | 47 | GC11M104942 | 11.3 |
| GSDMB | Protein Coding | 37 | GC17M039904 | 11.05 |
| GSDMC | Protein Coding | 33 | GC08M129705 | 8.68 |
| IL1B | Protein Coding | 49 | GC02M112829 | 7.83 |
| GZMB | Protein Coding | 46 | GC14M024630 | 7.28 |
| NLRP1 | Protein Coding | 45 | GC17M005499 | 7.12 |
| GSDMA | Protein Coding | 36 | GC17P039962 | 6.67 |
| GZMA | Protein Coding | 43 | GC05P055102 | 6.38 |
| NLRC4 | Protein Coding | 45 | GC02M032224 | 5.81 |
| CASP5 | Protein Coding | 44 | GC11M104995 | 5.66 |
| AIM2 | Protein Coding | 43 | GC01M159062 | 5.39 |
| PYCARD | Protein Coding | 43 | GC16M031201 | 5 |
| CASP3 | Protein Coding | 51 | GC04M184627 | 4.59 |
| DHX9 | Protein Coding | 41 | GC01P182839 | 4.04 |
| NLRP9 | Protein Coding | 35 | GC19M055711 | 4.04 |
| NAIP | Protein Coding | 41 | GC05M070968 | 3.95 |
| HMGB1 | Protein Coding | 45 | GC13M030456 | 3.95 |
| CASP8 | Protein Coding | 53 | GC02P201233 | 3.83 |
| FOXO3 | Protein Coding | 45 | GC06P108559 | 3.67 |
| IL18 | Protein Coding | 45 | GC11M112143 | 3.5 |
| APIP | Protein Coding | 41 | GC11M034854 | 3.5 |
| TXNIP | Protein Coding | 38 | GC01M145992 | 3.43 |
| GBP1 | Protein Coding | 42 | GC01M089052 | 2.9 |
| CASP6 | Protein Coding | 49 | GC04M109688 | 2.74 |
| NEK7 | Protein Coding | 43 | GC01P198156 | 2.7 |
| GJA1 | Protein Coding | 52 | GC06P121436 | 2.66 |
| P2RX7 | Protein Coding | 46 | GC12P123032 | 2.64 |
| TP53 | Protein Coding | 54 | GC17M007661 | 2.58 |
| MALT1 | Protein Coding | 48 | GC18P058671 | 2.57 |
| AGER | Protein Coding | 45 | GC06M032180 | 2.55 |
| TET2 | Protein Coding | 45 | GC04P105145 | 2.55 |
| EEF2K | Protein Coding | 47 | GC16P022217 | 2.52 |
| CD274 | Protein Coding | 46 | GC09P005450 | 2.52 |
| FGF21 | Protein Coding | 41 | GC19P048769 | 2.52 |
| CEBPB | Protein Coding | 45 | GC20P050190 | 2.48 |
| TFAM | Protein Coding | 45 | GC10P058385 | 2.48 |
| STK4 | Protein Coding | 50 | GC20P044966 | 2.43 |
| PRDM1 | Protein Coding | 46 | GC06P105993 | 2.43 |
| PRF1 | Protein Coding | 46 | GC10M070597 | 2.43 |
| MST1 | Protein Coding | 46 | GC03M049683 | 2.43 |
| ELAVL1 | Protein Coding | 43 | GC19M007958 | 2.43 |
| TREM2 | Protein Coding | 43 | GC06M043554 | 2.43 |
| HDAC6 | Protein Coding | 52 | GC0XP048801 | 2.36 |
| SQSTM1 | Protein Coding | 49 | GC05P179806 | 2.36 |
| IRF3 | Protein Coding | 47 | GC19M049659 | 2.36 |
| STING1 | Protein Coding | 35 | GC05M139476 | 2.36 |
| ZBP1 | Protein Coding | 39 | GC20M057603 | 2.31 |
| PECAM1 | Protein Coding | 42 | GC17M064319 | 2.27 |
| DDX3X | Protein Coding | 48 | GC0XP041333 | 2.11 |
| PRTN3 | Protein Coding | 45 | GC19P000840 | 1.87 |
| SERPINB1 | Protein Coding | 41 | GC06M002833 | 1.87 |
| NR1H2 | Protein Coding | 49 | GC19P050329 | 1.82 |
| CAMP | Protein Coding | 42 | GC03P048287 | 1.79 |
| MRE11 | Protein Coding | 42 | GC11M094636 | 1.77 |
| PARP1 | Protein Coding | 50 | GC01M226360 | 1.75 |
| CTSG | Protein Coding | 46 | GC14M024573 | 1.75 |
| GBP5 | Protein Coding | 38 | GC01M089259 | 1.75 |
| NLRP7 | Protein Coding | 44 | GC19M054923 | 1.65 |
| MKI67 | Protein Coding | 45 | GC10M128096 | 1.64 |
| IL36G | Protein Coding | 39 | GC02P112973 | 1.54 |
| IL36B | Protein Coding | 35 | GC02M113022 | 1.54 |
| CPTP | Protein Coding | 31 | GC01P001633 | 1.52 |
| BNIP3 | Protein Coding | 43 | GC10M131966 | 1.47 |
| ANO6 | Protein Coding | 40 | GC12P045215 | 1.47 |
| FADD | Protein Coding | 48 | GC11P070203 | 1.43 |
| MEFV | Protein Coding | 45 | GC16M003522 | 1.43 |
| APOL1 | Protein Coding | 43 | GC22P036253 | 1.43 |
| TNF | Protein Coding | 52 | GC06P049352 | 1.38 |
| VIM | Protein Coding | 51 | GC10P017227 | 1.38 |
| CAPN1 | Protein Coding | 50 | GC11P065201 | 1.38 |
| JUN | Protein Coding | 50 | GC01M058780 | 1.38 |
| ALK | Protein Coding | 53 | GC02M029190 | 1.22 |
| SIRT1 | Protein Coding | 50 | GC10P067884 | 1.22 |
| BIRC3 | Protein Coding | 47 | GC11P102317 | 1.22 |
| BIRC2 | Protein Coding | 46 | GC11P102347 | 1.22 |
| UBE2D2 | Protein Coding | 46 | GC05P139526 | 1.22 |
| LY96 | Protein Coding | 43 | GC08P073991 | 1.22 |
| RIPK3 | Protein Coding | 43 | GC14M024336 | 1.22 |
| GLMN | Protein Coding | 41 | GC01M092246 | 1.22 |
| IRGM | Protein Coding | 39 | GC05P150846 | 1.22 |
| NLRP13 | Protein Coding | 35 | GC19M055892 | 1.22 |
| TUBB6 | Protein Coding | 42 | GC18P012307 | 0.84 |
| NOS2 | Protein Coding | 50 | GC17M027756 | 0.72 |
| NOS1 | Protein Coding | 50 | GC12M117208 | 0.72 |
| PYDC2 | Protein Coding | 24 | GC03P191461 | 0.72 |
| IFI16 | Protein Coding | 43 | GC01P158969 | 0.71 |
| AKT1 | Protein Coding | 55 | GC14M104769 | 0.59 |
| EGFR | Protein Coding | 55 | GC07P055019 | 0.59 |
| TP63 | Protein Coding | 49 | GC03P189598 | 0.59 |
| ATF6 | Protein Coding | 48 | GC01P161766 | 0.59 |
| IRF1 | Protein Coding | 47 | GC05M132440 | 0.59 |
| IRF2 | Protein Coding | 45 | GC04M184387 | 0.59 |
| POP1 | Protein Coding | 41 | GC08P098117 | 0.59 |
| ORMDL3 | Protein Coding | 41 | GC17M039921 | 0.59 |
| MDM2 | Protein Coding | 54 | GC12P068808 | 0.5 |
| BTK | Protein Coding | 54 | GC0XM101349 | 0.5 |
| NFKB1 | Protein Coding | 53 | GC04P102501 | 0.5 |
| STAT3 | Protein Coding | 53 | GC17M042313 | 0.5 |
| BCL2 | Protein Coding | 52 | GC18M063123 | 0.5 |
| TLR2 | Protein Coding | 52 | GC04P153684 | 0.5 |
| ANXA2 | Protein Coding | 49 | GC15M060347 | 0.5 |
| IL1RN | Protein Coding | 49 | GC02P115722 | 0.5 |
| BECN1 | Protein Coding | 47 | GC17M042810 | 0.5 |
| CD14 | Protein Coding | 46 | GC05M140631 | 0.5 |
| GSTO1 | Protein Coding | 45 | GC10P104235 | 0.5 |
| IL13 | Protein Coding | 45 | GC05P132656 | 0.5 |
| CHI3L1 | Protein Coding | 44 | GC01M203148 | 0.5 |
| PANX1 | Protein Coding | 44 | GC11P094128 | 0.5 |
| LRPPRC | Protein Coding | 43 | GC02M043850 | 0.5 |
| CXCL8 | Protein Coding | 42 | GC04P073740 | 0.5 |
| IL13RA2 | Protein Coding | 42 | GC0XM115003 | 0.5 |
| IL32 | Protein Coding | 41 | GC16P004487 | 0.5 |
| BST2 | Protein Coding | 40 | GC19M017403 | 0.5 |
| GPER1 | Protein Coding | 38 | GC07P001247 | 0.5 |
| LYST | Protein Coding | 38 | GC01M235661 | 0.5 |
| CLEC5A | Protein Coding | 35 | GC07M141927 | 0.5 |
