# Supplementary material for: Prediction of Survival and Tumor Microenvironment Infiltration Based on Pyroptosis-Related lncRNAs in Pancreatic Cancer
Source: Dis Markers. 2022 Dec 30;2022:5634887. doi: 10.1155/2022/5634887 (PMC9822759; doi:10.1155/2022/5634887)
Supplement: Supplementary Materials — Figure S1: verification of the risk signature in entire set. (a) Grouping. (b) Scatter plot. (c) Heat map. (d) Survival analysis. (e) ROC curves. ROC: receiver operating characteristic. Figure S2: survival analyses of clinical subgroups. (a) Age ≤ 65. (b) Age > 65. (c) Female. (d) Male. (e) Grade 1-2. (f) Grade 3-4. (g) Stage I-II. (h) Stage III-IV. (i) T1-2. (j) T3-4. (k) N0. (l) N1-3. Figure S3: correlation of risk score with immune cells. (a) Activated NK cell. (b) M0 macrophage. (c) M1 macrophage. (d) M2 macrophage. (e) Resting mast cell. (f) Naive B cell. (g) Plasma cell. (h) CD8 T cell. (i) Activated memory CD4 T cell. (j) Regulatory T cell. (k) Gamma delta T cell. Table S1: gene list of 121 pyroptosis-related genes. Table S2: a total of 294 pyroptosis-related lncRNAs in TCGA. Table S3: seven pyroptosis-related lncRNAs involved in the risk signature. [file 5634887.f1.zip › Table S2.docx]

Table S2**:** A total of 294 pyroptosis-related lncRNAs in TCGA.

| ID |
| --- |
| OVOL1-AS1 |
| AC092171.5 |
| AC083841.1 |
| AC060766.4 |
| AC055822.1 |
| SUGT1P4-STRA6LP |
| ZBED3-AS1 |
| AP000696.1 |
| AC005089.1 |
| AC008514.1 |
| AC027228.2 |
| ZNF710-AS1 |
| AC022098.1 |
| AC037198.2 |
| AC010280.2 |
| PPP1R26-AS1 |
| LINC02489 |
| AL117336.2 |
| MIR503HG |
| AC009159.3 |
| LINC02600 |
| AC020659.1 |
| AC093904.3 |
| LINC01354 |
| AL139260.1 |
| AL031985.3 |
| LINC01754 |
| MIR9-3HG |
| AC010320.3 |
| WT1-AS |
| Z84723.1 |
| AC134043.2 |
| ST3GAL5-AS1 |
| LINC01150 |
| SENCR |
| SH3PXD2A-AS1 |
| AC005104.1 |
| C22orf34 |
| FAM27E3 |
| AC104653.2 |
| AC002091.1 |
| AC012020.1 |
| AL445524.1 |
| AC103746.1 |
| AL137186.2 |
| AP000894.4 |
| AL445471.1 |
| AL023653.1 |
| AC009812.4 |
| LINC02361 |
| AC087672.2 |
| SOX21-AS1 |
| AL355388.1 |
| DCST1-AS1 |
| AC138028.4 |
| FGF14-AS2 |
| AC079921.2 |
| ZMIZ1-AS1 |
| AL109615.3 |
| LINC01914 |
| AP005233.2 |
| AC080013.5 |
| AC109446.3 |
| LINC01124 |
| GHRLOS |
| LINC02562 |
| AP000345.2 |
| AC020663.2 |
| TONSL-AS1 |
| AC004921.1 |
| AC090948.3 |
| LINC02038 |
| AC099329.2 |
| AC011899.2 |
| AL391069.3 |
| AC093278.2 |
| AL161935.3 |
| AC012317.1 |
| HOXB-AS4 |
| AC002480.2 |
| LINC02323 |
| AC069209.1 |
| LINC02313 |
| AC067945.3 |
| AL109976.1 |
| AL645608.2 |
| LINC01374 |
| AC022509.1 |
| AL021807.1 |
| KDM7A-DT |
| AC090114.2 |
| AC011944.1 |
| AC099518.2 |
| LINC02593 |
| MIR155HG |
| LINC00847 |
| AC020658.5 |
| AC010904.2 |
| TMEM254-AS1 |
| AC243829.1 |
| LINC00857 |
| AC106876.1 |
| AC009948.3 |
| LINC02041 |
| LINC02595 |
| AC005332.4 |
| AL034374.1 |
| LINC01736 |
| LINC00628 |
| AL355601.1 |
| AC011899.3 |
| AL132800.1 |
| AC245128.3 |
| AC010175.1 |
| AL020997.3 |
| AC006033.2 |
| AC068580.2 |
| AC002091.2 |
| AC001226.1 |
| PCAT7 |
| U62317.2 |
| LINC01160 |
| AC005332.6 |
| LINC01827 |
| PAN3-AS1 |
| AC009690.2 |
| AC092171.3 |
| AC145098.1 |
| AC009065.5 |
| STARD4-AS1 |
| AC012645.3 |
| AP002954.1 |
| AP000439.2 |
| FLG-AS1 |
| GTSCR1 |
| AC093001.1 |
| AC055720.2 |
| COL4A2-AS1 |
| MIAT |
| AC083862.2 |
| AC138305.1 |
| AFAP1-AS1 |
| AC025048.4 |
| AL365181.2 |
| AC019254.1 |
| MIR194-2HG |
| C20orf197 |
| AL606534.1 |
| AC007546.1 |
| C1QTNF1-AS1 |
| AC010247.2 |
| CRNDE |
| AC021242.3 |
| AC005696.3 |
| AP001574.1 |
| LINC01978 |
| PCAT19 |
| DIAPH2-AS1 |
| AC005281.1 |
| AL133371.2 |
| AC009495.1 |
| SMIM25 |
| AC027682.6 |
| SNHG26 |
| AC002480.1 |
| SLCO4A1-AS1 |
| AP003774.1 |
| AP005131.2 |
| MEG9 |
| LINC02126 |
| AC090559.1 |
| AP000255.1 |
| TRPC7-AS1 |
| NEXN-AS1 |
| AC084876.1 |
| LINC01133 |
| TRAF3IP2-AS1 |
| AL022322.1 |
| LINC01833 |
| CASC19 |
| VAC14-AS1 |
| LINC02004 |
| DIRC3 |
| AP003559.1 |
| LINC01814 |
| AC023301.1 |
| AC007786.1 |
| AP001107.9 |
| AC012213.4 |
| AC006272.1 |
| AC006449.5 |
| AC007255.1 |
| AC078850.1 |
| AC007336.2 |
| AC009065.2 |
| LINC02518 |
| LINC00973 |
| FAM225A |
| LINC02285 |
| LINC02104 |
| AL390208.1 |
| AC096733.2 |
| LINC00092 |
| AC022706.1 |
| FOXD3-AS1 |
| AL137026.1 |
| LINC00519 |
| AC093904.2 |
| AC010615.2 |
| AC116351.1 |
| AL049555.1 |
| LINC02256 |
| AL807752.4 |
| AL365181.3 |
| CASC9 |
| AC138207.5 |
| AC027097.2 |
| AL355312.3 |
| AC105219.1 |
| AC005332.2 |
| AC007728.2 |
| LHX1-DT |
| AC009093.6 |
| LRRC8C-DT |
| AC243960.1 |
| AL606489.1 |
| C9orf139 |
| GUSBP11 |
| AL162595.1 |
| TRG-AS1 |
| AL118505.1 |
| TP53TG1 |
| AC253576.2 |
| AC078923.1 |
| AC004865.2 |
| BX470102.1 |
| AL356215.1 |
| MIR200CHG |
| AC007376.2 |
| AL049836.1 |
| ITGA9-AS1 |
| SATB1-AS1 |
| AC008870.3 |
| LINC01857 |
| AL583722.1 |
| AC108860.2 |
| AC005062.1 |
| AC005498.2 |
| AL162724.2 |
| AC104964.1 |
| AC027097.1 |
| PCED1B-AS1 |
| AC104653.1 |
| AC004687.1 |
| AP000812.1 |
| AC004990.1 |
| CARD8-AS1 |
| APTR |
| AC112496.1 |
| AC087501.4 |
| C6orf99 |
| ZNF236-DT |
| LINC01480 |
| AC127502.2 |
| TRIM31-AS1 |
| AC025154.2 |
| LINC00589 |
| ACTA2-AS1 |
| AC002059.1 |
| AC009119.1 |
| AC024941.2 |
| DNM3OS |
| LINC02391 |
| AC018682.1 |
| MEG3 |
| AC099518.4 |
| AC019186.1 |
| AC080013.3 |
| LINC02084 |
| AC008771.1 |
| LINC02158 |
| SYNPR-AS1 |
| LINC01091 |
| AC008750.1 |
| AP000238.1 |
| AC015660.1 |
| AC093904.4 |
| AC092807.2 |
| AC008972.2 |
| AC021188.1 |
| ZNF582-AS1 |
| LINC00852 |
| AF111169.3 |
| AL606834.2 |
