# Supplementary material for: Prediction of Survival and Tumor Microenvironment Infiltration Based on Pyroptosis-Related lncRNAs in Pancreatic Cancer
Source: Dis Markers. 2022 Dec 30;2022:5634887. doi: 10.1155/2022/5634887 (PMC9822759; doi:10.1155/2022/5634887)
Supplement: Supplementary Materials — Figure S1: verification of the risk signature in entire set. (a) Grouping. (b) Scatter plot. (c) Heat map. (d) Survival analysis. (e) ROC curves. ROC: receiver operating characteristic. Figure S2: survival analyses of clinical subgroups. (a) Age ≤ 65. (b) Age > 65. (c) Female. (d) Male. (e) Grade 1-2. (f) Grade 3-4. (g) Stage I-II. (h) Stage III-IV. (i) T1-2. (j) T3-4. (k) N0. (l) N1-3. Figure S3: correlation of risk score with immune cells. (a) Activated NK cell. (b) M0 macrophage. (c) M1 macrophage. (d) M2 macrophage. (e) Resting mast cell. (f) Naive B cell. (g) Plasma cell. (h) CD8 T cell. (i) Activated memory CD4 T cell. (j) Regulatory T cell. (k) Gamma delta T cell. Table S1: gene list of 121 pyroptosis-related genes. Table S2: a total of 294 pyroptosis-related lncRNAs in TCGA. Table S3: seven pyroptosis-related lncRNAs involved in the risk signature. [file 5634887.f1.zip › Table S3.docx]

Table S3. Seven pyroptosis-related lncRNAs involved in the risk signature.

| Gene | Coef |
| --- | --- |
| AC083841.1 | 0.32379802 |
| AC090114.2 | -0.514170677 |
| AC005332.6 | -0.00468968 |
| PAN3-AS1 | -0.076270242 |
| LINC01133 | 0.009627904 |
| AC087501.4 | -1.40433337 |
| AC015660.1 | 0.202042065 |
