# Supplementary figures and images for: Prediction of Survival and Tumor Microenvironment Infiltration Based on Pyroptosis-Related lncRNAs in Pancreatic Cancer
Source: Dis Markers. 2022 Dec 30;2022:5634887. doi: 10.1155/2022/5634887 (PMC9822759; doi:10.1155/2022/5634887)

**a**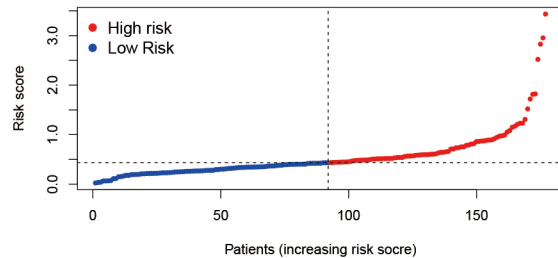**b**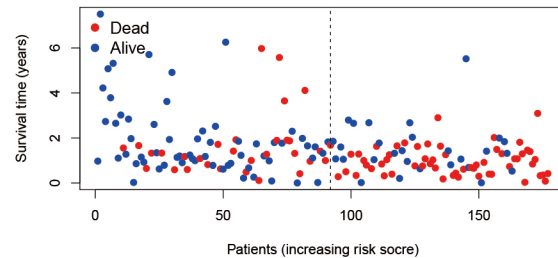**c**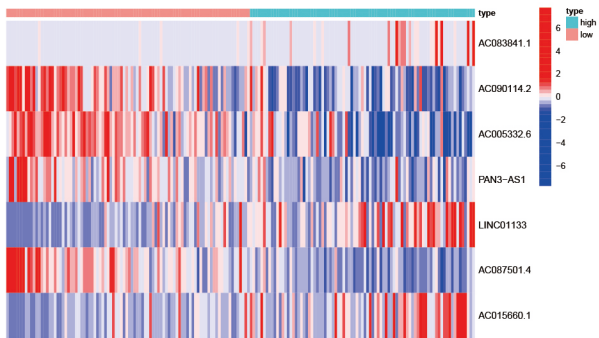**d**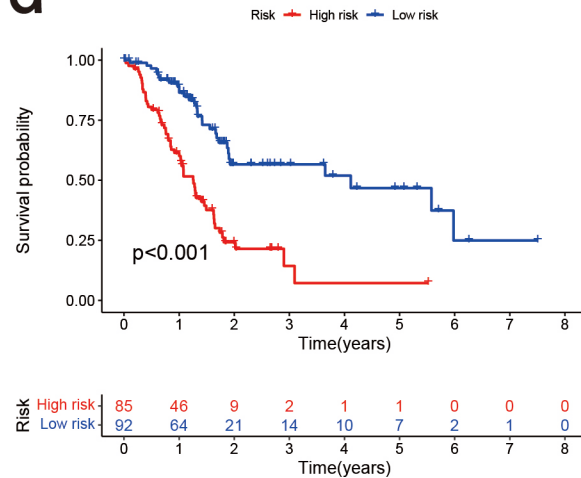**e**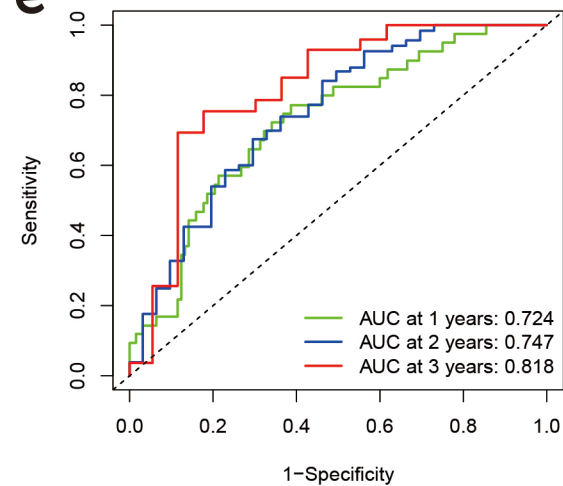

Supplement: Supplementary Materials — Figure S1: verification of the risk signature in entire set. (a) Grouping. (b) Scatter plot. (c) Heat map. (d) Survival analysis. (e) ROC curves. ROC: receiver operating characteristic. Figure S2: survival analyses of clinical subgroups. (a) Age ≤ 65. (b) Age > 65. (c) Female. (d) Male. (e) Grade 1-2. (f) Grade 3-4. (g) Stage I-II. (h) Stage III-IV. (i) T1-2. (j) T3-4. (k) N0. (l) N1-3. Figure S3: correlation of risk score with immune cells. (a) Activated NK cell. (b) M0 macrophage. (c) M1 macrophage. (d) M2 macrophage. (e) Resting mast cell. (f) Naive B cell. (g) Plasma cell. (h) CD8 T cell. (i) Activated memory CD4 T cell. (j) Regulatory T cell. (k) Gamma delta T cell. Table S1: gene list of 121 pyroptosis-related genes. Table S2: a total of 294 pyroptosis-related lncRNAs in TCGA. Table S3: seven pyroptosis-related lncRNAs involved in the risk signature. [file 5634887.f1.zip › Figure S1.pdf]

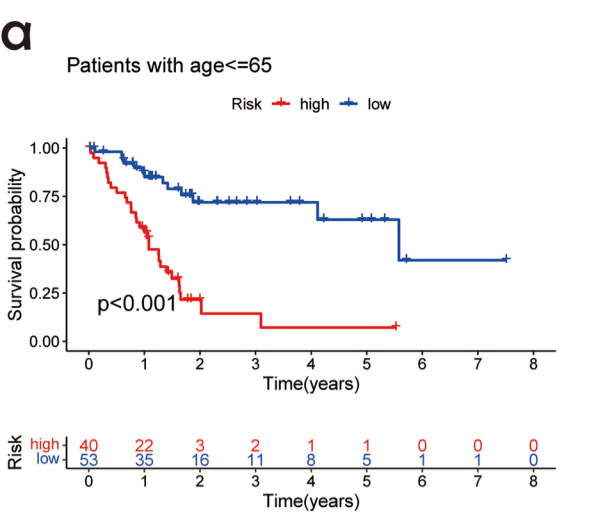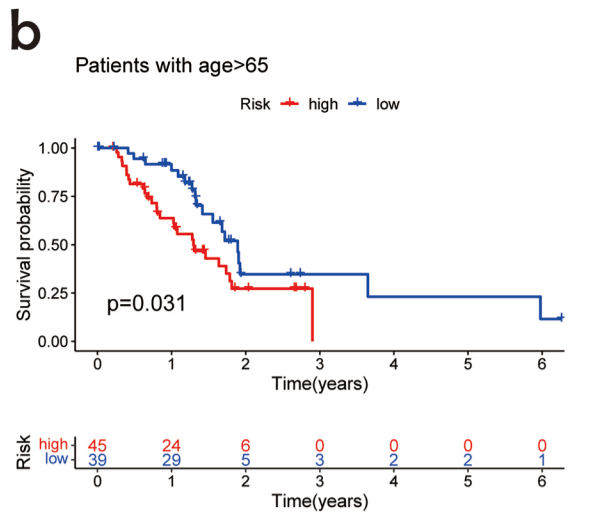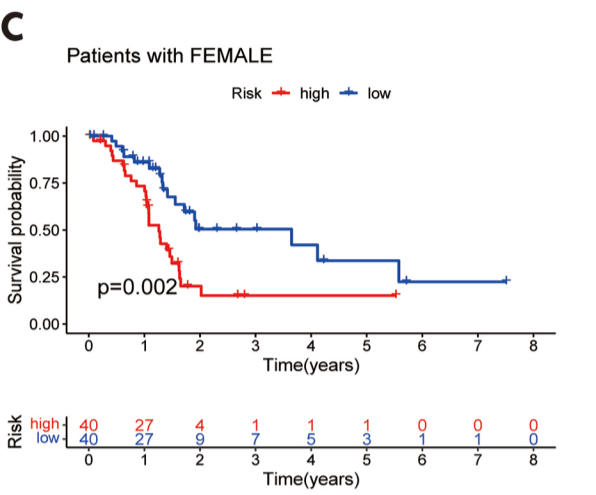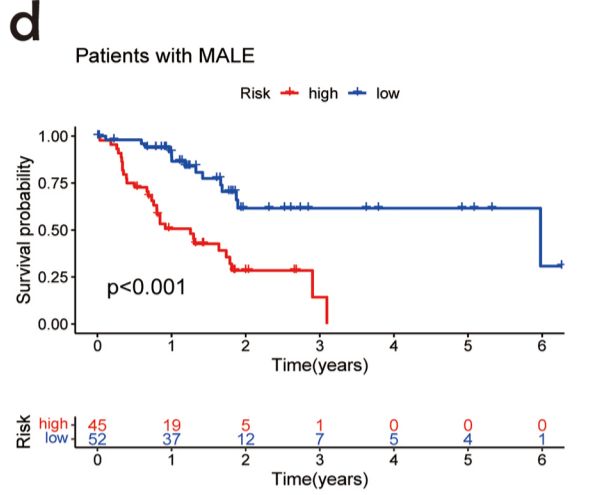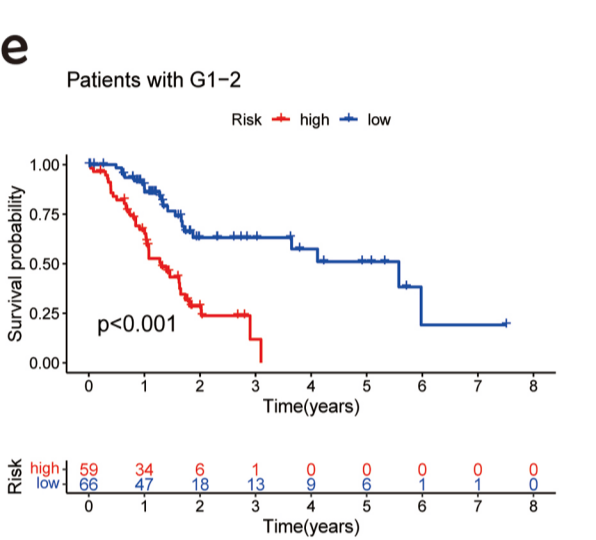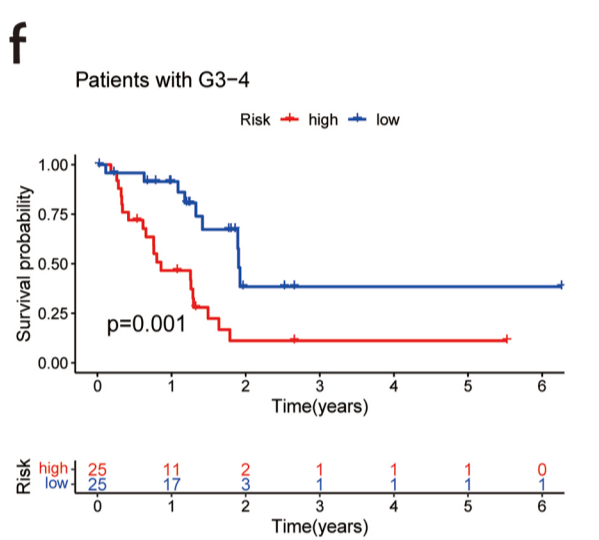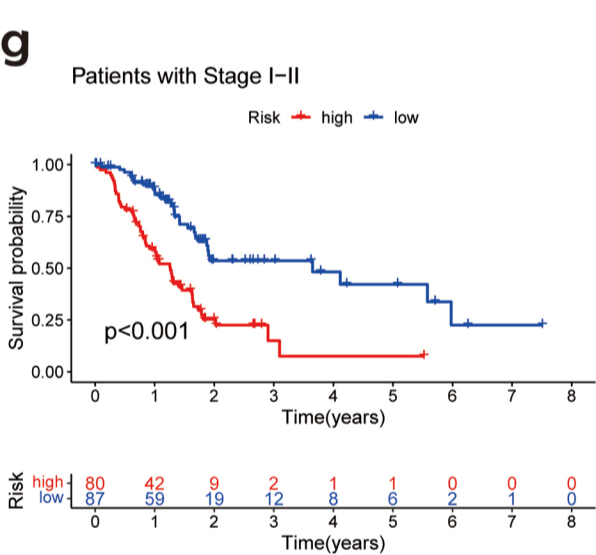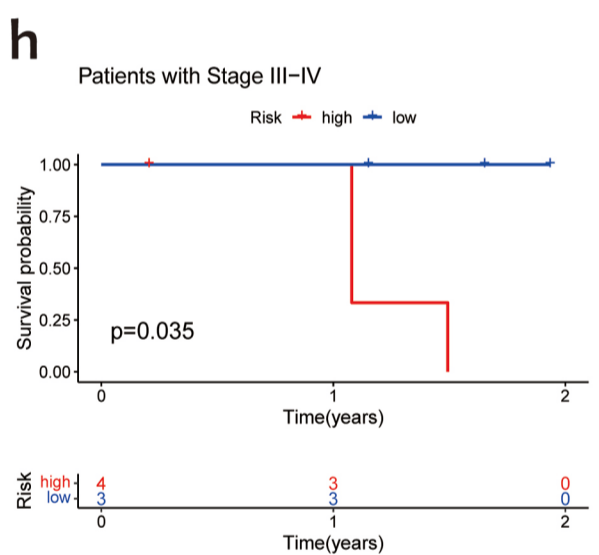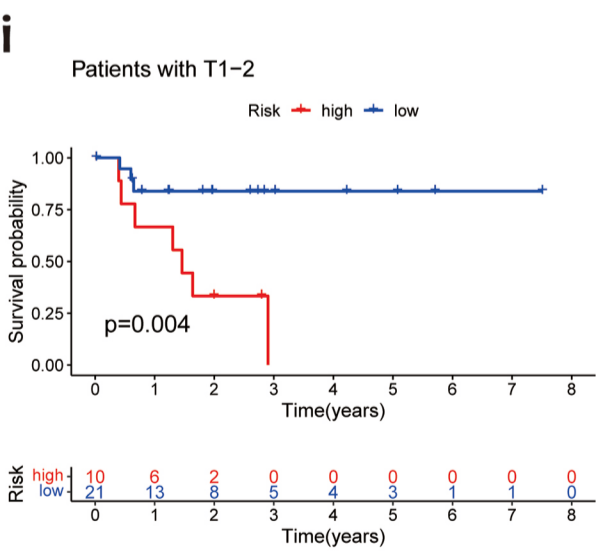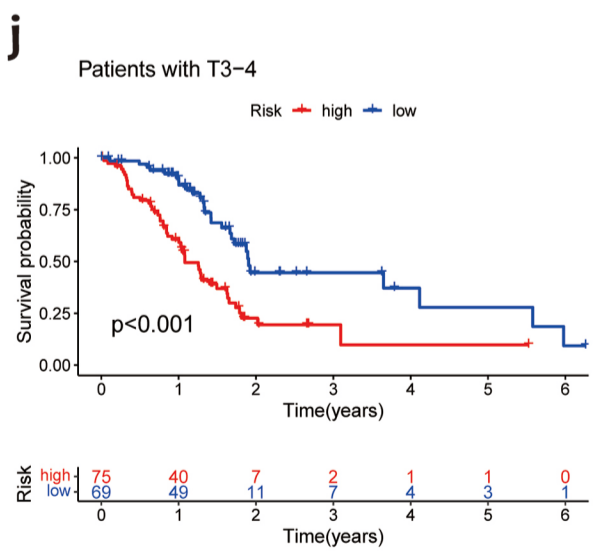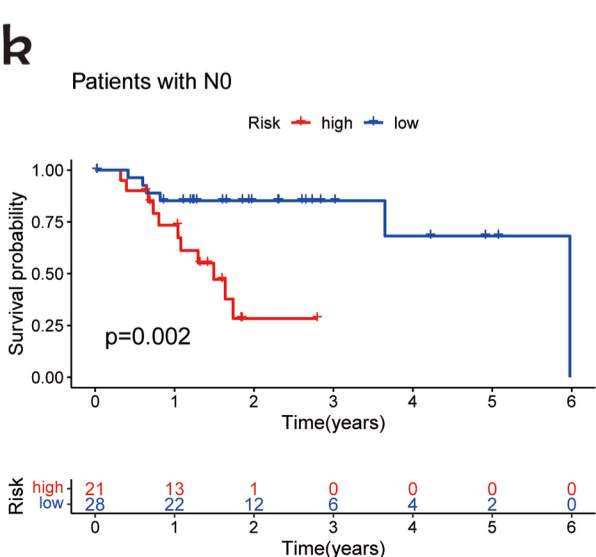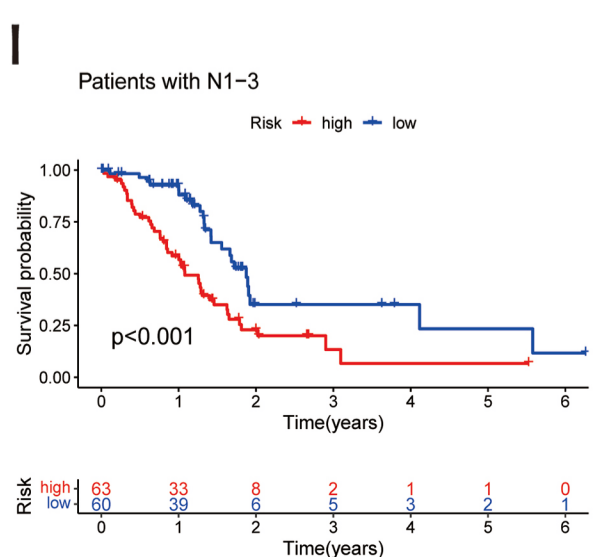

Supplement: Supplementary Materials — Figure S1: verification of the risk signature in entire set. (a) Grouping. (b) Scatter plot. (c) Heat map. (d) Survival analysis. (e) ROC curves. ROC: receiver operating characteristic. Figure S2: survival analyses of clinical subgroups. (a) Age ≤ 65. (b) Age > 65. (c) Female. (d) Male. (e) Grade 1-2. (f) Grade 3-4. (g) Stage I-II. (h) Stage III-IV. (i) T1-2. (j) T3-4. (k) N0. (l) N1-3. Figure S3: correlation of risk score with immune cells. (a) Activated NK cell. (b) M0 macrophage. (c) M1 macrophage. (d) M2 macrophage. (e) Resting mast cell. (f) Naive B cell. (g) Plasma cell. (h) CD8 T cell. (i) Activated memory CD4 T cell. (j) Regulatory T cell. (k) Gamma delta T cell. Table S1: gene list of 121 pyroptosis-related genes. Table S2: a total of 294 pyroptosis-related lncRNAs in TCGA. Table S3: seven pyroptosis-related lncRNAs involved in the risk signature. [file 5634887.f1.zip › Figure S2.pdf]

**a**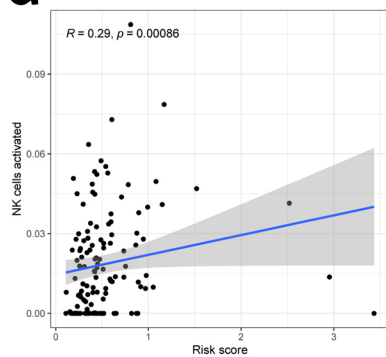**b**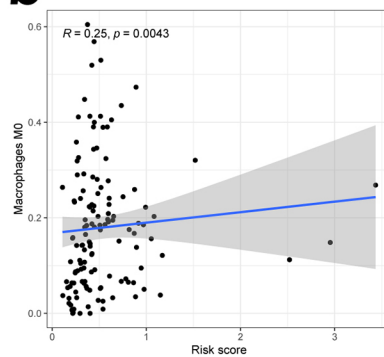**c**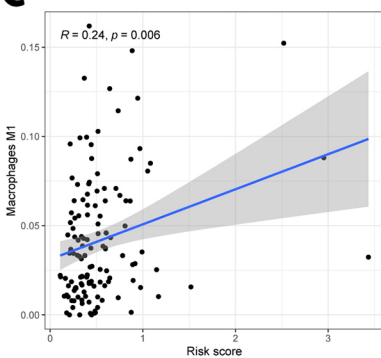**d**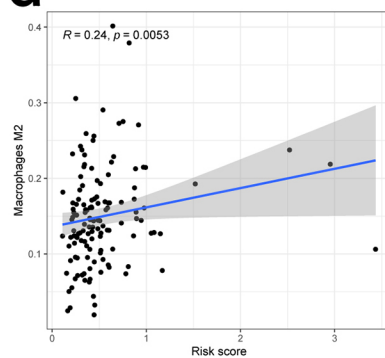**e**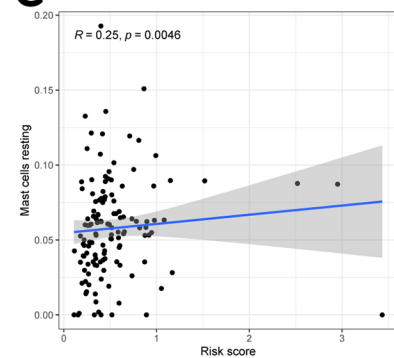**f**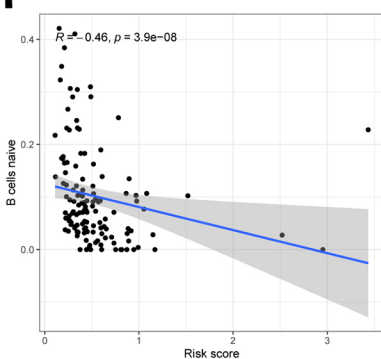**g**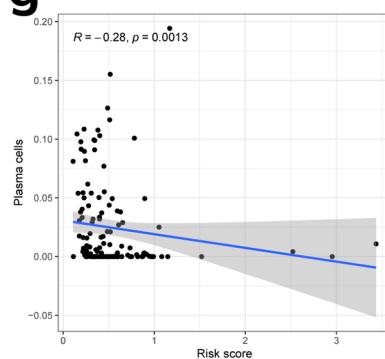**h**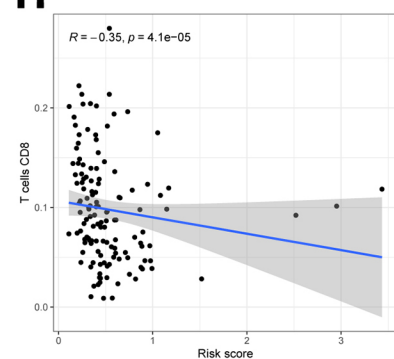**i**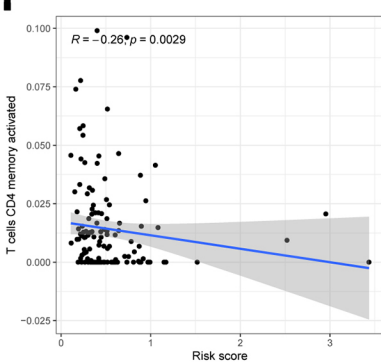**j**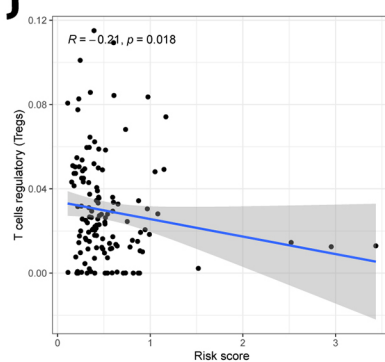**k**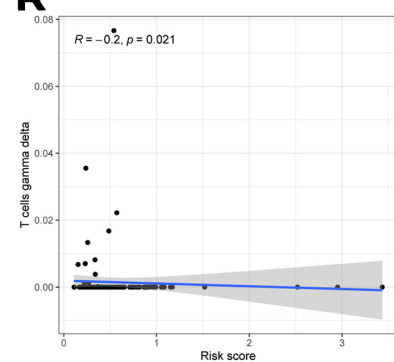

Supplement: Supplementary Materials — Figure S1: verification of the risk signature in entire set. (a) Grouping. (b) Scatter plot. (c) Heat map. (d) Survival analysis. (e) ROC curves. ROC: receiver operating characteristic. Figure S2: survival analyses of clinical subgroups. (a) Age ≤ 65. (b) Age > 65. (c) Female. (d) Male. (e) Grade 1-2. (f) Grade 3-4. (g) Stage I-II. (h) Stage III-IV. (i) T1-2. (j) T3-4. (k) N0. (l) N1-3. Figure S3: correlation of risk score with immune cells. (a) Activated NK cell. (b) M0 macrophage. (c) M1 macrophage. (d) M2 macrophage. (e) Resting mast cell. (f) Naive B cell. (g) Plasma cell. (h) CD8 T cell. (i) Activated memory CD4 T cell. (j) Regulatory T cell. (k) Gamma delta T cell. Table S1: gene list of 121 pyroptosis-related genes. Table S2: a total of 294 pyroptosis-related lncRNAs in TCGA. Table S3: seven pyroptosis-related lncRNAs involved in the risk signature. [file 5634887.f1.zip › Figure S3.pdf]
